# Primary Small Intestinal Leiomyosarcoma Presenting With Small Bowel Perforation: A Case Report

**DOI:** 10.7759/cureus.74352

**Published:** 2024-11-24

**Authors:** Basim J Busada, Sreekala Sreehari, Salman Heydari Khajehpour, Ashish Massey, Mohammed Aboubeirah

**Affiliations:** 1 Department of General Surgery, University Hospitals Dorset NHS Foundation Trust, Bournemouth, GBR; 2 Department of Pathology, University Hospitals Dorset NHS Foundation Trust, Bournemouth, GBR

**Keywords:** ct scan, gastrointestinal surgery, gist, histology, leiomyosarcoma, perforation, preoperative diagnosis, rare tumor, sarcoma, small intestine

## Abstract

A ​small intestinal leiomyosarcoma (LMS) is a rare malignancy that accounts for a small proportion of small bowel tumors and is often difficult to diagnose early due to its variable symptoms. This case report describes a 75-year-old female patient, previously suspected to have a gastrointestinal stromal tumor (GIST), presenting to the ED with abdominal pain and elevated inflammatory markers. A CT scan was performed which revealed a perforated mass in the distal ileum with surrounding collection. Consequently, she underwent laparoscopic washout, bowel resection, and primary anastomosis. Postoperatively, she developed paralytic ileus, which resolved with conservative management, and she was eventually discharged postoperative day 14. Histological and immunohistochemical analysis confirmed the diagnosis of an LMS, showing smooth muscle actin positivity, strong caldesmon labeling, and focal desmin expression with negative c-kit and S-100. The diagnosis of LMSs can be difficult, often misdiagnosed as GISTs due to overlapping features. Imaging studies, including CT scans, are valuable but not definitive. This case highlights the necessity of recognizing primary small intestinal LMSs as a potential cause of bowel perforation. Due to the rarity of LMSs and their potential for misclassification, accurate diagnosis and multidisciplinary management are crucial. Given the high risk of recurrence and metastasis, particularly due to perforation, long-term follow-up is also recommended.

## Introduction

Malignancies of the small bowel account for less than 5% of all gastrointestinal cancers [1]. Among these, leiomyosarcomas (LMSs) are one of the rarest, representing less than 2% [2]. An LMS is a subtype of sarcoma that arises from smooth muscles and affects various parts of the small bowel including the jejunum (32%), ileum (25.2%), and duodenum (12.6%) [1]. Most cases are asymptomatic and pose a challenge in diagnosis with the most common presentations being nonspecific abdominal pain or discomfort, anemia, bowel obstruction, or bleeding [2]. In this article, we present an extremely rare presentation of an LMS as a bowel perforation.

## Case presentation

**Figure 1:**
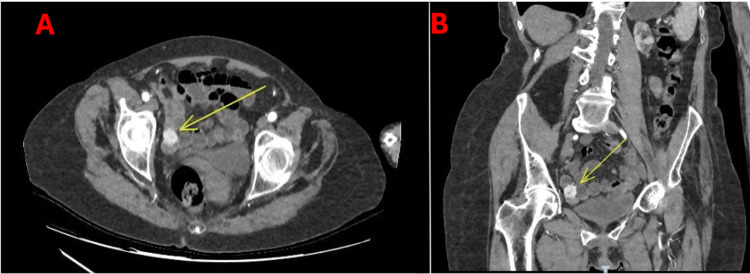
Contrast-enhanced (arterial phase) computed tomography (CT) scan before perforation A: Axial view; B: Coronal view. The yellow arrow indicates a 2 cm avidly enhancing small bowel mass initially suspected to be a GIST. GIST: Gastrointestinal stromal tumor

A 75-year-old female patient presented with a four-day history of worsening lower abdominal pain, malaise, and anorexia. She had a history of atrial fibrillation and a previous cerebrovascular accident. Three months prior, she presented with lower GI bleeding and was suspected to have a small bowel gastrointestinal stromal tumor (GIST) (Figure 1) but was managed conservatively due to her frailty. 

On presentation, she was hemodynamically stable with an irregular heartbeat of 117 but apyrexial and maintaining good blood pressure. On examination, her abdomen was soft but tender in the lower abdomen with percussion tenderness and guarding on deep palpation. 

Her blood tests revealed a significantly elevated CRP of 398 and an elevated amylase level of 121, but a normal white blood cell count (Table 1).

**Table 1:** Laboratory investigations Lab results on admission showing elevated neutrophil, CRP, and amylase levels.

Parameters	Values	Reference value
WBC	9.6	4.0- 10.0 (10*9/L)
Neutrophil count	8.2	2.0- 7.0 (10*9/L)
Lymphocyte count	0.6	1.0- 3.0 (10*9/L)
CRP	398	0-9 (mg/l)
Amylase	121	0-100 (U/L)

**Figure 2:**
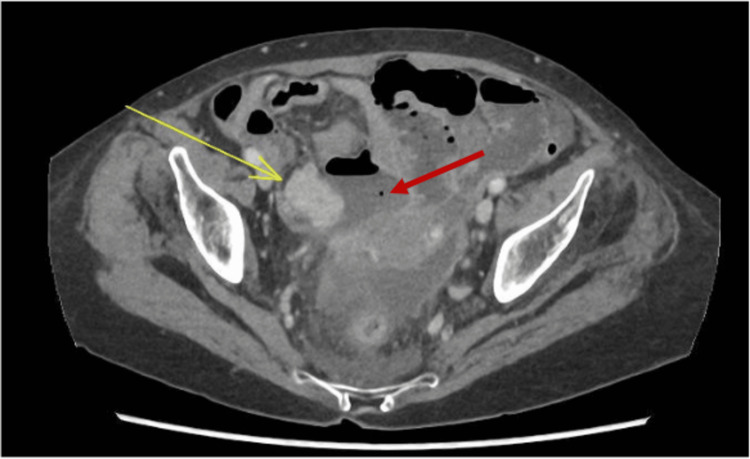
Contrast-enhanced computed tomography (CT) scan post perforation The yellow arrow indicates a homogeneously hyperenhancing mass (LMS) at the mid-distal ileum measuring approximately 31 x 20 mm in size (compared to 20mm in the previous scan) and the red arrow indicates gas and fluid-containing collection measuring approximately 43 x 49 mm.

The computed tomography (CT) scan revealed a 31x20 mm homogeneously hyper enhancing mass in the mid-distal ileum, which has grown from 20 mm in comparison to the CT scan three months ago. There was also a 43 x 49 mm gas and fluid-containing collection adjacent to it which extended superiorly in the small bowel mesentery and posteriorly to a larger 87 x 28 x 45 mm ill-defined fluid collection in the Douglas pouch, with associated peritoneal enhancement. These findings, along with the peritoneal enhancement, fat stranding, and thickening of the nearby small bowel, were indicative of a small bowel perforation (Figure 2).

Based on the clinical picture and investigations, a plan was made for a diagnostic laparoscopy and small bowel resection. The estimated mortality rate, calculated using the National Emergency Laparotomy Audit (NELA) risk prediction model, was found to be 16.64%. ​​​The mortality and risk of surgery were discussed with the patient and her relatives, and she opted for surgery. ​​Intra-operatively, a large pelvic abscess/phlegmon was identified, drained and washed out. 50 cm from the ileocecal valve was a 4 cm inflamed small bowel with a perforation. This was exteriorized, resected along with mesentery and associated lymph nodes and a side to side, isoperistaltic, hand-sewn anastomosis was formed. The patient was admitted to the intensive care unit postoperatively for monitoring, where she developed paralytic ileus that resolved with conservative management. She was discharged on postoperative day 14. 

**Figure 3:**
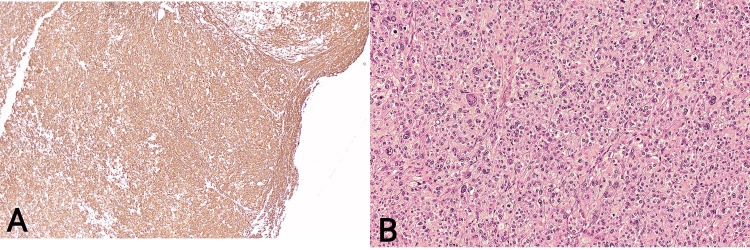
Histology appearance of the tumor A: Caldesmon showing strong positivity, confirming leiomyosarcoma (H, ×4); B: Sheets of cells with epithelioid morphology, confirming epithelioid leiomyosarcoma Grade 3 (HE, ×10)

The histology report identified a 30 x 30 x 10 mm, well-circumscribed, perforated tumor located in the muscularis propria and subserosa, with direct tumor perforation through the serosal surface but no evidence of lymphovascular or perineural invasion. Immunohistochemical staining showed that the tumor was negative for DOG-1, c-kit, S100, Melan-A, SOX10, TFE3, HMB45, ERG, CD34, and synaptophysin. There was weak positivity for SMA and focal positivity for MNF116. Additional staining revealed strong diffuse labeling for caldesmon, focal expression of desmin, and occasional weak to moderate cytokeratin labeling. Examination of the resected lymph nodes (0/13) showed no involvement. Together, the tumor's morphology and immunoprofile are consistent with a diagnosis of a primary small intestinal LMS (Figure 3). 

The patient has been monitored in accordance with the sarcoma protocol and discussed at the regional multidisciplinary team (MDT) meeting. The recommended management plan includes regular follow-up with CT scans every 4-6 months, due to the increased risk of intraperitoneal and systemic metastasis associated with the perforation. To date, both a CT scan and a PET scan have shown no evidence of local or distant metastasis. Given the absence of metastatic disease, surgical resection alone was deemed sufficient, and chemotherapy is not indicated at this time. The patient remains disease-free 10 months post-treatment. 

## Discussion

Diagnosis of LMSs can be very challenging with the possibility of misdiagnosing them as GISTs being very common, as occurred in our case [2,3]. This is particularly relevant in the post-‘GIST era,’ where advances in diagnostic tools, especially the immunostaining of KIT protein by Hirota et al. in 1998, led to the reclassification of most stromal tumors as GISTs rather than LMSs [4]. This was so extensive that the WHO classified LMSs to be extremely rare, with no significant data on demographic, clinical, or gross features of this tumor [5]. By 2012, there were only 28 cases of small bowel LMSs recorded in the literature [5]. 

One of the tools that can be used to differentiate them is CT scans. According to Megibow et al., CT scans are useful tools in the diagnosis of these lesions. However, it poses a challenge since LMSs do not have a particular pattern. They can be exophytic, lobulated, or irregular-shaped which limits the overall benefit of CT in determining the location of the tumor [6]. In our case, it was initially misdiagnosed as GIST based on the CT picture but that might also be due to its rarity. 

During the resection, we ensured complete removal of the mass with adequate safety margins, including the mesentery and lymph nodes. It is worth noting that routine resection of lymph nodes is rarely required as both GISTs and LMSs infrequently exhibit lymph node metastases [7,8]. Despite their rare incidence, they have been reported multiple times in the literature [8]. In 2009, Agaimy and Wünsch reported two cases of lymph node involvement among 210 patients with GISTs, representing 1% [9]. In our case, 13 lymph nodes were resected, and they all came back negative.

Clinically, most LMSs are asymptomatic with patients mainly presenting with progression of disease. They commonly present as acute abdomen, anemia due to bleeding or intussusception [10]. According to Matsuda et al., who reviewed Japanese literature from 1980 to 1989, the frequency of LMS perforation was found to be 8.6% [11]. However, this was before the redefinition of LMSs by the WHO, and so we think the rate is much lower. 

We searched PubMed for LMSs presenting with perforation and identified two cases that fit these criteria. One of them was due to a large 20 cm LMS that had invaded an adjacent bowel resulting in its perforation [10]. So, unlike our case, this case perforated because of invasion of the LMS into the adjacent bowel, rather than a direct perforation of the LMS lesion. The other case was a perforated small bowel due to a uterine leiomyosarcoma metastasis to the small bowel [12]. Therefore, to the best of our knowledge, our ​case​​​ could be​​​​​​ the first-ever reported case of a primary LMS presenting with perforation. 

Finally, regarding prognosis and recurrence, LMSs carry a high risk of recurrence, ranging from 39% to 80%, with the likelihood of secondary metastases between 55% and 71% [13]. In the case of a perforated LMS, we suspect the risk of recurrence and/or metastasis to be higher due to the dissemination of cancer cells within the abdominal cavity [14]. Therefore, close follow-up will be required under the guidance of the multidisciplinary team [2]. 

## Conclusions

This case underlines an exceptionally rare presentation of a primary small intestinal LMS in the form of small bowel perforation, a complication previously undocumented in the literature. Small bowel LMSs are an extremely rare malignancy, often asymptomatic or presenting with nonspecific symptoms. These factors make their diagnosis not only challenging but also liable to misclassification (as seen in our case with an initial GIST diagnosis). Moreover, given the associated high risks for recurrence and metastasis, particularly with perforated LMSs, close postoperative follow-up and a multidisciplinary approach are recommended. Finally, this case highlights the limited understanding of LMSs and underscores the importance of differential diagnosis in rare gastrointestinal malignancies. 
